# Coming of Age: Targeting Cyclin K in Cancers

**DOI:** 10.3390/cells12162044

**Published:** 2023-08-11

**Authors:** Yi Xiao, Jixin Dong

**Affiliations:** Eppley Institute for Research in Cancer and Allied Diseases, Fred and Pamela Buffett Cancer Center, University of Nebraska Medical Center, Omaha, NE 68198, USA; yi.xiao@unmc.edu

**Keywords:** cyclin, CDK, Cyclin K, phosphorylation, cancer, inhibitor, degrader

## Abstract

Cyclins and cyclin-dependent kinases (CDKs) play versatile roles in promoting the hallmarks of cancer. Therefore, cyclins and CDKs have been widely studied and targeted in cancer treatment, with four CDK4/6 inhibitors being approved by the FDA and many other inhibitors being examined in clinical trials. The specific purpose of this review is to delineate the role and therapeutic potential of Cyclin K in cancers. Studies have shown that Cyclin K regulates many essential biological processes, including the DNA damage response, mitosis, and pre-replicative complex assembly, and is critical in both cancer cell growth and therapeutic resistance. Importantly, the druggability of Cyclin K has been demonstrated in an increasing number of studies that identify novel opportunities for its use in cancer treatment. This review first introduces the basic features and translational value of human cyclins and CDKs. Next, the discovery, phosphorylation targets, and related functional significance of Cyclin K-CDK12/13 complexes in cancer are detailed. This review then provides a summary of current Cyclin K-associated cancer studies, with an emphasis on the available Cyclin K-targeting drugs. Finally, the current knowledge gaps regarding the potential of Cyclin K in cancers are discussed, along with interesting directions for future investigation.

## 1. Introduction

In the 1980s, the discovery of cyclins and cyclin-dependent kinases (CDKs) significantly influenced the realm of biology. The term “cyclins” was originally coined by Tim Hunt in his study of sea urchins, due to their constant and cyclical fluctuation pattern during synthesis and degradation throughout the cell cycle [1]. At nearly the same time, CDKs were characterized by Paul M. Nurse as a group of proteins that propel cell cycle transition in an orderly manner by forming complexes with the corresponding cyclins [2]. 

To date, 46 human cyclins containing cyclin box domains (CBD) or cyclin box-like domains have been identified, among which 29 cyclins have been found to be the direct activators of 21 human CDKs [3]. Cyclins and CDKs are highly conserved, from yeasts to mammals; according to their major functions, they can be generally categorized as cell cycle or transcriptional cyclins/CDKs [4,5]. 

The cell cycle cyclins/CDKs regulate different phases of the cell cycle. Specifically, Cyclin C-CDK3 regulates the G0 phase. Cyclin D-CDK4/6 and Cyclin E-CDK2 regulate the G1 phase. Cyclin A-CDK2 regulates the S phase. The G2 phase is regulated by Cyclin A-CDK1. The M phase is regulated by Cyclin B-CDK1, etc. [6,7]. 

The transcriptional cyclins/CDKs phosphorylate the carboxy-terminal domain (CTD) of RNA polymerase II to regulate gene expression. For example, Cyclin C-CDK8/19 mediates the pre-initiation of transcription, Cyclin H-CDK7 regulates the initiation of transcription, Cyclin T-CDK9 and Cyclin K-CDK12/13 regulate transcription elongation, Cyclin L-CDK11 regulates the termination of transcription, etc. [7,8].

The dysregulation of cyclins and CDKs is a common feature in cancer [9]. Cyclins/CDKs contribute to the hallmarks of cancer in various ways, including promoting cell cycle progression, facilitating cell migration, inhibiting apoptosis, increasing DNA damage repair, regulating cancer stem cell self-renewal, etc. [9,10,11]. 

Targeting cyclins/CDKs impairs cancer growth, reduces therapeutic resistance, and activates anti-tumor immunity [12,13,14]. Given the critical role of cyclins/CDKs in cancer, cyclin/CDK-targeting therapies have been the focal point of cancer research for many years [12,15,16]. Notably, the FDA has approved the following four CDK4/6 inhibitors: abemaciclib, palbociclib, ribociclib, and trilaciclib, which are used for the treatment of hormone receptor (HR)-positive and human epidermal growth factor receptor 2 (HER2)-negative breast cancer. Furthermore, trilaciclib has been used to lower the rate of chemotherapy-induced myelosuppression [16,17]. 

Recently, multiple CDK inhibitors have been developed and examined in clinical trials (Table 1). Of all these CDK-associated interventional cancer studies, CDK4/6 inhibitors account for the major proportion. Along with the FDA-approved CDK4/6 inhibitors, other CDK4/6 inhibitors (i.e., dalpiciclib, lerociclib, SPH4336, TQB3616, TQB3303, XZP-3287, HS-10342, PRT3645, FCN-437c, BPI-1178, GLR2007, and CS3002) have been tested in a variety of malignancies. In addition, a more specific CDK4 inhibitor (i.e., PF-07220060) and two more broad CDK4/6 inhibitors (i.e., FLX925 and ETH-155008) are under examination in the context of certain advanced solid tumors and hematologic malignancies. CDK2, CDK7, and CDK9 are also popular CDK targets in cancers, including CDK2-specific inhibitors (i.e., PF-07104091, INX-315, and INCB123667), CDK7-specific inhibitors (i.e., samuraciclib, SY-5609, Q901, and XL102), and CDK9-specific inhibitors (i.e., enitociclib, AZD4573, KB-0742, and GFH009), as well as more promiscuous CDK inhibitors (e.g., dinaciclib, fadraciclib, roniciclib, voruciclib, and riviciclib hydrochloride). Additionally, CDK8 and CDK19 are preferentially targeted together (i.e., RVU120 and BCD-115) and have emerged as a new group of CDK targets for advanced malignancies.

The broad application of CDK inhibitors in cancer treatment raises challenges as well as opportunities. Most CDK inhibitors target the ATP binding pocket, yet the CDK family of proteins share a highly conserved catalytic domain [18]. A major concern is posed by drug toxicity resulting from the low selectivity of the CDK inhibitors. Despite the biological significance of the cyclins, they do not possess catalytic sites for inhibitor development. Therefore, targeting cyclins has long been difficult. 

The advancement of targeted protein degradation technologies heralds a new era for molecular therapies that greatly expands the range of druggable proteins, increases target specificity, allows for low-dose interventions, and reduces the development of drug resistance [19]. Targeted protein degradation strategies harness the natural proteasomal or lysosomal degradation machinery to eliminate, rather than just inhibit, the proteins of interest [19,20]. Of all the various targeted protein degradation technologies (e.g., PROTACs, molecular glue, LYTAC, and AUTAC), the proteolysis-targeting chimeras (PROTACs) and molecular glues have demonstrated the greatest therapeutic potential for cancers [19,20,21]. 

PROTACs and molecular glue function similarly in that they bring the targeted protein in proximity to the E3 ligase and utilize the ubiquitin-proteasome system for protein degradation [22]. They are different in terms of their chemical structures, in that PROTACs are bivalent compounds, while molecular glue is a monovalent compound [22]. Currently, in clinical trials for cancer treatment, there are quite a few PROTACs and molecular glues targeting estrogen receptors and androgen receptors (e.g., BCL-XL, BTK, STAT3, IKZF2, and IKZF1/3) [20,21]. In recent years, degraders against CDKs (CDK2, CDK4, CDK6, CDK8, CDK9, and CDK12) have been highly sought-after and have demonstrated promising results [23,24]. Moreover, degraders against previously deemed “undruggable” cyclins (Cyclin K and Cyclin D1) have been discovered and developed [25,26]. Here, considering the tremendous wave of targeted protein degradation developments in cancer therapeutics, and their use for specific purposes, this review elaborates on the features of Cyclin K, highlights the important role of Cyclin K in cancers, summarizes the characteristics of the Cyclin K degraders that have been discovered so far, and addresses some of the as-yet uncovered areas for future investigation.

## 2. Find the Partners: This Takes Time and Effort

### 2.1. Cyclin K: The Regulatory Subunit of CDK12 and CDK13, Not CDK9

Human *Cyclin K* cDNA was first identified in 1998 as a cell-cycle progression restoration (CPR) gene, used to restore the growth of *Saccharomyces cerevisiae* that lacked the yeast G1 cyclins Cln1, Cln2, and Cln3 [27]. It consists of 580 amino acids with two N-terminal cyclin box domains, mediating the binding of CDKs and a C-terminal proline-rich region [28]. Cyclin K was found to co-immunoprecipitate with RNA polymerase II and to possess strong kinase activity against CTD in vitro; therefore, it was considered a new member of the transcriptional cyclins group [27]. During the early years after its discovery, Cyclin K was believed to be a regulatory subunit, along with cyclin T1, cyclin T2a, and cyclin T2b, of CDK9 in the positive transcription elongation factor b (P-TEFb) complex [29,30,31]. However, the unbiased mass spectrometry studies did not identify Cyclin K from the CDK9 interactome, which cast doubts on the validity of the Cyclin K-CDK9 partnership [32,33]. Later, *Drosophila* Cyclin K (dCyclin K) was found to associate with purified dCDK12 (ortholog to human CDK12 and CDK13), yet dCDK9 was not detected in the purification complex [34]. In 2011, a key study from Blazek et al. demonstrated that human Cyclin K was a 70-kDa protein, not previously mentioned 40-kDa protein that formed a complex with CDK9; its confirmed partners were CDK12 and CDK13 [35]. This concept has been widely accepted since the findings of Blazek et al. were reported in 2011.

### 2.2. CDK12 and CDK13: The Partner Kinases for Cyclin K, Not Cyclin L

At the turn of the 21st century, human *CDK12* (*CRKRS*) and *CDK13* (*CDC2L5*) were identified through a cDNA screening that sought CDC2 (ortholog to human CDK1)-related kinases that are critical for early embryogenesis in sea urchins [36]. CDK12 and CDK13 consist of 1490 and 1512 amino acids, respectively, and display a centered kinase domain with arginine/serine-rich (RS) domains at the N-terminus and proline-rich regions at the N- and C-termini [28]. CDK12 and CDK13 share 43% of similarities in terms of overall sequence identity and >93% of similarities in their kinase domain [28,37]. CDK12 and CDK13 belong to the transcriptional CDKs due to their strong kinase activity against CTD in vitro [34]. 

It was believed in the early years that CDK12 and CKD13 formed complexes with L-type cyclins through tag-mediated co-immunoprecipitation [38,39]. However, a more stringent “pull-down” assay, followed by mass spectrometry analysis, identified dCyclin K, not dCyclin L, as the cyclin associated with dCDK12 in the cells [34]. This result was further confirmed by Blazek et al., who demonstrated Cyclin K to be the only regulatory subunit for CDK12 and CDK13 in human cells [35]. It took researchers approximately 10 years to corroborate the partnership between Cyclin K and CDK12/13. Consequently, exciting discoveries were in store for the next decade.

## 3. Investigate the Functions from a Phosphorylation Standpoint

### 3.1. Cyclin K-CDK12 and Cyclin K-CDK13 Phosphorylate CTD to Regulate Transcription

**Figure 1:**
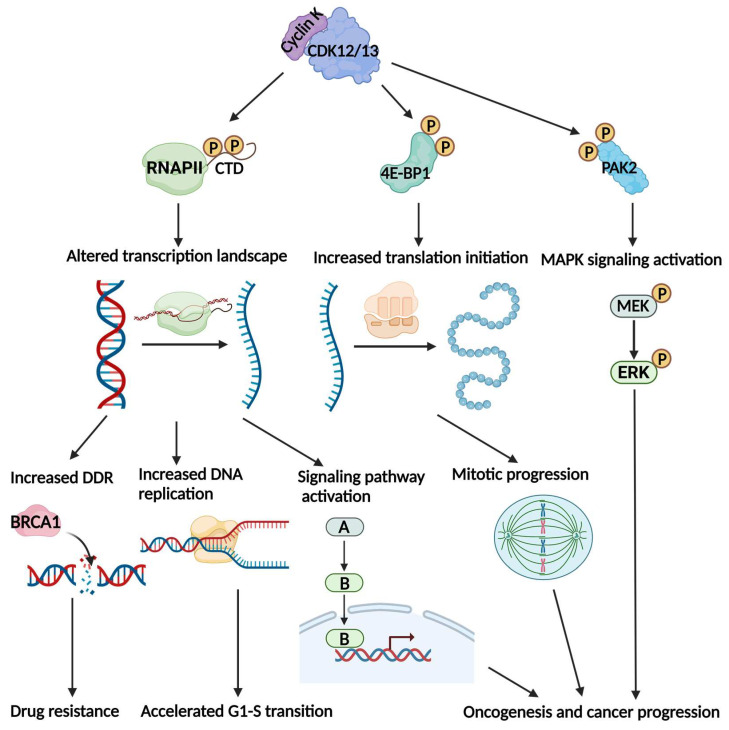
The Cyclin K-CDK12/13 complexes affect important biological processes and contribute to cancer development by phosphorylating different targets. Cyclin K-CDK12/13 phosphorylates the carboxy terminal domain (CTD) of RNA polymerase II to control transcription elongation and mRNA processing. This gives rise to the increased transcription of DNA damage repair genes and DNA replication genes, as well as multiple genes that are involved in the activation of cancer signaling. Cyclin K-CDK12/13 also phosphorylates 4E-BP1 to facilitate translation initiation. The mitotic regulators are the translation-specific targets in this process, allowing for accelerated mitotic progression. The p21-activated kinase 2 (PAK2) target is another phosphorylation target of Cyclin K-CDK12. By phosphorylating PAK2, Cyclin K-CDK12 activates the mitogen-activated protein kinase (MAPK) signaling pathway. As a result, Cyclin K-CDK12/13 promotes oncogenesis, cancer progression, and drug resistance.

CDK12 and CDK13 are the most well-known CTD kinases that require Cyclin K for their kinase activities. In this section, we detail the phosphorylation targets of Cyclin K-CDK12/13 complexes and their related functional significance in cancer (Figure 1).

The CTD, or the “tail” of RNA polymerase II, is composed of 52 tandem repeats of the consensus amino acid sequence Y^1^S^2^P^3^T^4^S^5^P^6^S^7^ found in mammals. Additionally, this domain is subject to multi-site phosphorylation on serine, threonine, and tyrosine [40]. Moreover, changes in CTD phosphorylation patterns orchestrate the transcription cycle [40]. The Cyclin K-CDK12 and Cyclin K-CDK13 complexes preferentially phosphorylate the Ser2 and Ser5 of the CTD and, to a much lesser extent, Ser7 in vitro [37,41,42]. Different methods have also validated the phosphorylation of Ser2 and Ser5 by Cyclin K-CDK12 in the cells [34,35,43]. Interestingly, although Ser5 is a more predominant site for phosphorylation, Ser2 is a more specific site. In one study, it was shown that the kinase-dead CDK12 or CDK13 failed to phosphorylate Ser2, yet they succeeded in phosphorylating Ser5 in vitro [37]. Ser2 phosphorylation is known as an important signal for transcription elongation and mRNA processing [44,45,46]; these studies suggested the ways in which Cyclin K-CDK12/13 complexes affected transcription. 

Studies by Yu et al. in 2015 and Qiu et al. in 2023 showed that Cyclin K-CDK12 phosphorylated LEO1, a subunit of polymerase II-associated factor 1 complex (PAF1C), to promote the phosphorylation of the CTD on Ser2, as well as processive transcription elongation [47,48]. Notably, compared to the moderate changes made by single CDK12 or CDK13 inhibition, the inhibition of CDK12/13 by a dual CDK12/13 inhibitor THZ531 dramatically reduced the phosphorylation of the CTD, decreased global RNA polymerase II processivity, and reduced the rate of transcription elongation, indicating a functional redundancy between CDK12 and CDK13 [49]. Moreover, multiple RNA splicing factors were found in association with CDK12 and CDK13. For example, the dual inhibition of CDK12/13 disrupted the interaction between the splicing factor SF3B1 and RNA polymerase II, resulting in the decreased phosphorylation of Ser2 and widespread intron retention [42,50,51]. Therefore, the Cyclin K-CDK12/13-mediated phosphorylation of CTD is shown to play an essential role in transcription control.

CDK12 regulates the alternative splicing of the last exon of genes with long transcripts and many exons. Additionally, CDK12 represses the usage of intronic polyadenylation sites to prevent the production of truncated mRNA [51,52,53]. This feature makes DNA damage-response genes especially susceptible to the inhibition of CDK12, providing a possible point of weakness in CDK12 and, thus, a new opportunity for cancer therapies. 

In the early 2010s, Blazek et al. used an expression microarray to explore the pool of genes that are transcriptionally altered by Cyclin K or CDK12 [35]. They observed a strong correlation with the genes affected by both Cyclin K and CDK1. The network analysis identified *Breast Cancer gene 1* (*BRCA1)* as the primary target of Cyclin K. *BRCA1*, along with other DNA damage response genes (e.g., *ATR*, *FANCI*, and *FANCD2*) were validated as the targets of Cyclin K/CDK12 via RT-PCR and yet, surprisingly, CDK13 depletion failed to change the expression of these genes. 

*BRCA1* and *BRCA2* are the central players of homologous recombination. *BRCA* loss (also known as “BRCAness”) is a signifier for sensitivity to poly-ADP ribose polymerase inhibitors (PARPi) [54]. A genome-wide synthetic lethal shRNA screening first disclosed the finding that CDK12 deficiency conferred vulnerability to PARPi in high-grade serous ovarian adenocarcinoma [55]. Later, the synthetic lethality achieved by the combination of the pharmaceutical inhibition of CDK12/13 or the downregulation of Cyclin K and PARPi was further demonstrated in EWS/FLI-positive Ewing sarcoma, triple-negative breast cancer, and castration-resistant prostate cancer [56,57,58]. Currently, the deleterious mutation of CDK12 is being used as a biomarker for PARPi therapies for prostate cancer patients in clinical trials (i.e., NCT04030559 and NCT02952534). In addition, homologous recombination deficiency predicted an improved response to DNA-damaging agents, and CDK12 depletion was shown to render sensitivity to a few DNA-damaging chemotherapeutics, including cisplatin, irinotecan, and doxorubicin [57,59].

DNA replication genes belong to another group of genes that are highly reliant on Cyclin K-CDK12 activity [60]. It was shown that CDK12 inhibition diminished the processivity of RNA polymerase II on a subset of DNA replication genes, leading to a decrease in the rate of transcription elongation of the target genes. As a result, Cyclin K depletion and/or CDK12 inhibition suppressed the expression of DNA replication genes (e.g., *MCM10*, *TOPBP1*, *ORC2*, etc.) and hindered the G1-to-S phase transition. Given that uncontrolled cell proliferation is an important hallmark of cancer and may be triggered by accelerated cell cycle progression [6,61], targeting Cyclin K-CDK12 can potentially hold back malignant transformation.

Cyclin K-CDK12 contributes to cancer development by transcriptionally regulating the expression of the oncogene *MYC* and promoting the cancer signaling pathways, including the noncanonical nuclear factor kappa B (NF-κB) pathway, the canonical Wnt pathway (WNT-β-catenin), and the erythroblastic leukemia viral oncogene/phosphoinositide 3-kinase/protein kinase B (ErbB–PI3K–AKT) pathway. *MYC* family members belong to the “super-transcription factors” that drive tumor progression through a variety of means [62]. CDK12 was found to initiate cleavage, while polyadenylation factor CstF77 was found to facilitate the optimal 3′-end processing of MYC pre-mRNA, thereby directly regulating the transcription of *MYC* [45]. The dual CDK12/13 inhibitor, THZ531, may also effectively suppress *MYC* expression [63]. Furthermore, Cyclin K-CDK12 can control the noncanonical NF-κB pathway by increasing the transcription of its central activators, MAP3K14 and NF-κB2 [64]. Depleting CDK12 or inhibiting Cyclin K-CDK12 with the compound 919278 decreased the MAP3K14 and NF-κB2 transcripts, resulting in insufficient translation of the NF-κB-inducing kinase (NIK) and p100 proteins for noncanonical NF-κB pathway activation. CDK12 also increased the expression of WNT ligands (i.e., WNT1 and WNT3), as well as the adaptor protein IRS1, at mRNA levels, thereby augmenting the WNT-β-catenin and ErbB–PI3K–AKT signaling pathways for breast cancer initiation and trastuzumab resistance [65].

### 3.2. Cyclin K-CDK12 and Cyclin K-CDK13 Phosphorylate Translation Machinery Regulate Translation

The first study known to associate CDK12/13 to translation initiation was published by Coordes et al. in 2015, who indicated that the kinase subunit of the carboxy-terminal domain kinase I (*Ctk1*, the yeast ortholog to human CDK12 and CDK13) was required for effective translation initiation [66]. Ctk1 depletion attenuated cap-dependent translation initiation and resulted in the decreased formation of 80S initiation complexes [66]. In mammalian cells, Cyclin K-CDK12 was found to directly phosphorylate the mRNA 5′ cap-binding repressor 4E-BP1 on Ser65 and Thr70 after primed phosphorylation by mTORC1 on Thr36 and Thr46 [67]. According to their model, the sequential phosphorylation of 4E-BP1 by mTORC1 and Cyclin K-CDK12 released 4E-BP1 from the cap-binding complex and enabled the initiation of translation. Recently, another study by Wu et al. in 2023 showed that CDK13 directly interacted with several translation machinery components and phosphorylated 4E-BP1 on Thr46 and eIF4B on Ser422 [68]. Moreover, CDK13 depletion significantly suppressed polysome assembly. Interestingly, an early study in 2007 by Röther et al. suggested that Ctk1 was also involved in translation elongation via phosphorylating the ribosome subunit Rps2 on Ser238 in yeast [69]. Whether similar mechanisms work in mammals warrants further investigation.

To determine the subset of genes that are specifically regulated by Cyclin K-CDK12 at translational levels, Choi et al. combined Ribo-Seq with RNA-Seq to screen the “translation-only” target mRNAs [67]. They identified a cluster of genes that are critical for mitotic progression, such as *CENP-B*, *NDC80*, *NUF2*, etc. Notably, Cyclin K or CDK12 deficiency led to severe chromosome misalignment, the activation of the spindle assembly checkpoint (SAC), and spindle pole detachment. Given that for years, mitosis has been viewed as a promising cancer target [70], targeting Cyclin K or CDK12 may provide an alternative strategy for mitotic-based anti-cancer therapy. Intriguingly, polysome profiling analysis showed that CDK13 was required for *MYC* translation [68]. Together, the current findings indicate that *MYC* is regulated by Cyclin K-CDK12/13 at both the transcriptional and translational levels.

### 3.3. Cyclin K-CDK12 Phosphorylates PAK2 to Regulate the MAPK Signaling Pathway

Dysregulation of the mitogen-activated protein kinase (MAPK) signaling pathway has been shown to contribute to carcinogenesis and therapeutic resistance in cancers [71]. CDK12 was found to activate the MAPK signaling pathway via the phosphorylation of p21-activated kinase 2 (PAK2) on Thr134 and Thr169 [72]. Phosphorylation-deficient PAK2 failed to activate the MEK-ERK axis and exerted a dominant negative effect on gastric cancer growth, both in vitro and in vivo.

## 4. A Shift in Perspective: Cyclin K as a Cancer Target

### 4.1. Cyclin K Expression in Normal Conditions and in Cancer

Compared to the “catalytic subunit” of the Cyclin K-CDK12/13 complexes, the role played by Cyclin K in cancers is largely underexplored. Researchers are more inclined to study CDKs rather than cyclins because CDKs possess defined enzymatic activity and an active center. However, with an increasing number of studies demonstrating the promising druggability of Cyclin K in recent years, it is timely to redirect this focus toward understanding the involvement of Cyclin K in cancer biology.

Under normal physiological conditions, Cyclin K expression follows homeostatic regulation. In the developmental cycle, during the early embryonic stages of murine embryos, Cyclin K was detected in the embryonic region (E6.5), the ectoderm (E7.5), and the tissue and organs being formed (E8.5–E11.5) [35]. The expression of Cyclin K was revealed to increase in the murine embryonic stem (ES) cells, was reduced by cell differentiation and became undetectable three weeks after differentiation [73]. Murine ES cells have also been shown to express increased levels of Cyclin K compared to their differentiated derivatives or tissue-specific stem cells. In murine and human adult tissues, Cyclin K has been shown to be highly expressed in the testes, especially in the spermatogonial stem cells, but is only mildly expressed in the lungs, liver, stomach, and ovaries, and is barely detectable in the brain, heart, kidneys, and muscles [74]. Additionally, Cyclin K expression was elevated during liver regeneration after partial hepatectomy [75]. Nevertheless, pathological conditions occur when the homeostasis of Cyclin K expression is disrupted. For example, the knockout of Cyclin K in murine embryos led to embryonic lethality [35]. Furthermore, the ectopic expression of Cyclin K in adult tissue resulted in cancer development [74]. Indeed, oncogenes or cancer-promoting genes are often overexpressed in cancers and, in many cases, genes that are highly expressed and essential for embryonic development, but that are silenced and non-essential for adult life, are promising cancer targets [76]. Therefore, it is possible that Cyclin K is a prime target for targeted therapeutics in treating cancer.

**Figure 2:**
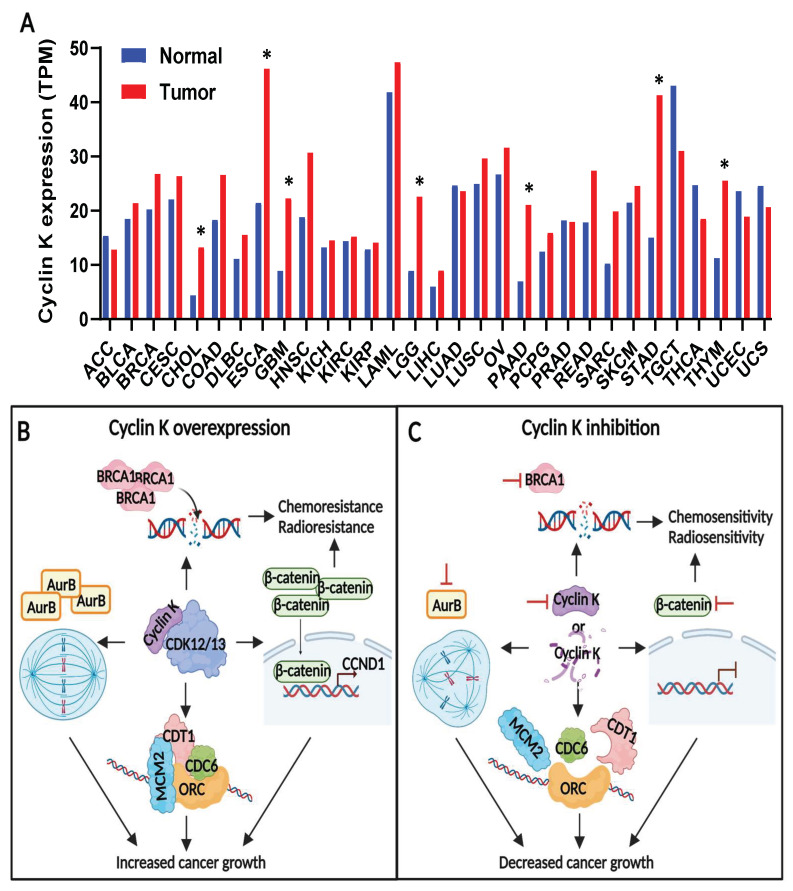
The expression and function of Cyclin K in cancers. (**A**) The expression of Cyclin K mRNA across different types of cancers is based on the TCGA and GTEx data. The expression levels of Cyclin K are shown as the logarithm to the base 2 of the number of transcripts per million. The average expression data was derived from GEPIA2 and the figure was created using Prism. An ANOVA was used to analyze the statistical significance of the original data. The asterisks (*p* < 0.01) indicate the various Cyclin K-overexpressed cancer types, including cholangiocarcinoma (CHOL), esophageal carcinoma (ESCA), glioblastoma (GBM), low-grade glioma (LGG), pancreatic adenocarcinoma (PAAD), stomach adenocarcinoma (STAD), and thymoma (THYM). (**B**) Cyclin K promotes the transcription of Aurora B (AurB), BRCA1, and β-catenin, and enhances the assembly of pre-replicative complex (pre-RC), thereby contributing to cancer growth and therapeutic resistance. (**C**) The genetic depletion or pharmaceutical degradation of Cyclin K attenuates the expression of AurB, BRCA1, and β-catenin, and also hinders pre-RC assembly. This impairs cancer growth and confers therapeutic vulnerability.

Based on the TCGA and GTEx data from the public cancer portal, GEPIA2 [77], Cyclin K mRNA expression is increased in several types of cancers, including cholangiocarcinoma, esophageal carcinoma, glioblastoma, low-grade glioma, pancreatic adenocarcinoma, stomach adenocarcinoma, and thymoma (Figure 2A). In addition, Cyclin K overexpression in cancers has been validated in cancer cell lines and cancer patient samples in the scientific literature. For example, Lei et al. observed that Cyclin K was overexpressed in various human cancer cell lines and in invasive breast ductal carcinoma samples [75]. They also saw an increased level of Cyclin K in late-stage breast cancer, compared to that in early-stage breast cancer. As shown in another study, Cyclin K expression was markedly enhanced in primary prostate cancer, and the increased expression of Cyclin K was associated with decreased biochemical recurrence-free survival [78]. They further pointed out that Cyclin K could be used as an independent biomarker for predicting biochemical recurrence-free survival for patients with prostate cancer. Similarly, Yao et al. observed elevated levels of Cyclin K in lung cancer from the lung adenocarcinoma tissue arrays. Their study also found that the increased expression of Cyclin K was associated with more advanced cancer stages [79]. Although the deep-sequencing technologies employed granted easy access to the genomic and transcriptomic data, little is known about how Cyclin K is overexpressed in cancers. More genomic and epigenetic studies are needed to unveil this mystery. 

Given that Cyclin K is vital for embryonic survival, another interesting field to examine is the role of Cyclin K in tissue development and disease control. Tissue-specific transgenic mouse models are likely to provide answers to these questions.

### 4.2. Cyclin K Contributes to Tumor Growth and Therapeutic Resistance in Cancers

Current Cyclin K-related cancer studies focus on investigating the role of Cyclin K in tumor growth and therapeutic resistance (Figure 2B,C). Having observed the increased expression of Cyclin K in human testicular cancer, Xiang et al. then examined the function of Cyclin K in testicular cancer cells [74]. They found that Cyclin K depletion dramatically reduced cancer cell proliferation. This result gave one of the first hints to the potential implication of Cyclin K in cancer. Later, Schecher et al. investigated the role of Cyclin K in prostate cancer. They found that Cyclin K was required for prostate cancer cell growth [78]. They also showed that Cyclin K deficiency resulted in chromosome misalignment, chromosome mis-segregation, and the formation of multinucleated cells. Their findings indicated that these mitotic defects might be a consequence of the Cyclin K depletion-mediated transcriptional downregulation of Aurora B. Additionally, the knockdown of Cyclin K suppressed lung cancer cell proliferation, both in vitro and in vivo [79]. By directly interacting with β-catenin, Cyclin K increased the protein level of β-catenin and promoted the expression of its target gene, *Cyclin D1*. It was further demonstrated that Cyclin D1 partially mediated the biological function of Cyclin K in lung cancer cells. Moreover, the genetic or pharmaceutical ablation of Cyclin K restricted colorectal cancer growth in cell line-derived xenograft (CDX) and patient-derived xenograft (PDX) models [80]. 

Chemical-mediated Cyclin K degradation (e.g., dCeMM2/3/4, 7f, or 7b) has been shown to impair the viability of various cancer cells [81,82]. In contrast, Lei et al. showed that inducing the expression of Cyclin K in the cancer cells stimulated cancer cell growth, both in vitro and in vivo, in a dose-dependent manner [75]. Lei et al. found that Cyclin K knockdown led to the dysregulation of pre-replicative complex (pre-RC) assembly, which then gave rise to G1-S arrest. Mechanistically speaking, Cyclin K-CDK12 phosphorylated Cyclin E1 on Ser366 to abrogate its interaction with CDK2 during G1. Due to the increased activity of Cyclin E1-CDK2 blocking the loading of pre-RC components (i.e., CDT1 and MCM2) onto the chromatin in G1, in turn, Cyclin K-CDK12 promoted pre-RC formation by disrupting the interaction between Cyclin E1 and CDK2 and ensured the transition of G1-S. Together, these studies demonstrate that Cyclin K contributes to cell proliferation in cancer by regulating the following: the mitotic process, pre-RC assembly, and the protein abundance of β-catenin.

The involvement of Cyclin K in the DNA damage response pathway offers great opportunities in the context of radiotherapy and chemotherapy for cancer treatment. For example, Yao et al. found that in lung cancer, Cyclin K depletion resulted in elevated DNA strand breaks and also rendered cells more sensitive to radiation-induced DNA damage [79]. Notably, upon radiation challenge, Cyclin K knockdown decreased the Cyclin D1-mediated recruitment of Rad51 to the DNA damage sites. Rad51 plays an important role in homologous recombination. Thus, these findings further demonstrate the role of Cyclin K in the β-catenin-Cyclin D1 axis-mediated DNA damage response. 

In another study, Sun et al. showed that the androgen receptor binds to the Cyclin K promotor to increase the expression of Cyclin K [58]. Therefore, the androgen deprivation therapy-mediated downregulation of Cyclin K offers therapeutic opportunities for PARPi, due to the attenuation of homologous recombination. Considering that castration resistance is common in prostate cancer patients receiving hormone therapy, PARPi may provide therapeutic avenues for this specific group of patients. The pharmaceutical degradation of Cyclin K by the molecular glue NCT02 also sensitized colorectal tumor spheroid culture cells, such as oxaliplatin and irinotecan, to chemotherapy [80]. The combination of a Cyclin K degrader (e.g., dCeMM2/3/4, PP-C8, or 7f) and DNA-damage agents (e.g., cisplatin, irinotecan, olaparib, or MK-8776) produced synergistic effects in several different types of cancer cells [81,82,83]. In addition, a genome-wide shRNA screening identified Cyclin K as a candidate for camptothecin resistance [84]; however, more research is needed to validate this result. 

While the participation of Cyclin K in immune therapy has not been explored, the research published by Wen et al. indicates its potential relevance [85]. Their research revealed that the CDK12/13 inhibitor SR4835 induced the immunogenic cell death of cancer cells by elevating endoplasmic reticulum stress and promoting dendritic cell activity. The combination of SR4835 and the programmed cell death protein 1 (PD-1) blockade increased the number as well as the activity of immune cells in tumors, resulting in tumor shrinkage in vivo. Despite being originally designed as a dual CDK12/13 inhibitor, SR4835 effectively degraded Cyclin K [57,80]. It is, therefore, possible that the anti-cancer immunity is activated, or partially activated, by the degradation of Cyclin K. Whether and in what way Cyclin K contributes to immune suppression are valuable questions that merit further examination.

### 4.3. Cyclin K Is a Druggable Target in Cancers

Interestingly, notwithstanding the relatively limited studies on Cyclin K, several Cyclin K-degrading drugs have been discovered and developed (Table 2), implying the high druggability of Cyclin K. Overall, these drugs can be categorized into the following three categories: (**1**) molecular glue degraders (e.g., HQ461, NCT02, and dCeMM2/3/4) [80,81,86], (**2**) PROTAC degraders (e.g., PP-C8 and 7f/7b) [82,83], and (**3**) Cyclin K-degrading CDK inhibitors (e.g., SR4835 and CR8) [25,80].

The molecular glue HQ461 was discovered after phenotype-based high-throughput screening for NRF2 inhibitors conducted by Lv et al. [86]. Knockout of the DDB1-CUL4-RBX1 E3 ubiquitin ligase complex components (DDB1 or RBX1) or the CDK12 mutation at the kinase domain (G731E or G731R) conferred resistance to HQ461. It was first speculated that CDK12 was the target of the DDB1-CUL4-RBX1-mediated ubiquitin-proteasome system. However, HQ461 only decreased the amount of CDK12 protein at a modest rate, and the complete depletion of CDK12 failed to phenocopy the effect of HQ461 on the cells. Lv et al. then questioned whether Cyclin K was affected by HQ461. They found that Cyclin K was more sensitive to HQ461 treatment than CDK12. Nevertheless, HQ461 did not alter the protein level of Cyclin K in cells with a CDK12 mutation (i.e., G731E or G731R). Lv et al. later demonstrated that HQ461 is the molecular glue that connects CDK12 and DDB1, possibly via the ATP-binding pocket of CDK12, and leads to the proteasomal degradation of Cyclin K. The loss of Cyclin K, in turn, reduced the stability of CDK12. 

The molecular glue NCT02 was discovered after a high-throughput screening for compounds with inhibitory activity against patient-derived colorectal tumor spheroid culture cells [80]. NCT02 exhibited a pronounced selectivity against tumor spheroid culture cells, compared to normal primary fibroblasts. Transcriptomic analysis followed by proteomic analysis identified Cyclin K-CDK12 as the target of NCT02. As seen in HQ461, NCT02 also acted as a molecular glue connecting CDK12 to DDB1 by binding at the ATP-binding site of CDK12 and occupying the pocket of DDB1. NCT02 triggered the proteasomal degradation of Cyclin K and indirectly affected the stability of CDK12. 

The molecular glues, dCeMM2, dCeMM3, or dCeMM4 (abbreviated as dCeMM2/3/4), were discovered from a comparative chemical screening of hyponeddylated versus neddylation-proficient cells, conducted to identify the cytotoxic molecules that are functionally linked to cullin-RING ligases [81]. Quantitative expression proteomics revealed that dCeMM2/3/4 dramatically degraded Cyclin K and, to a lesser extent, CDK12 and CDK13. Similarly, dCeMM2/3/4 connected CDK12 and CDK13 to the CRL4B E3 ligase complex, resulting in the ubiquitination and degradation of Cyclin K. Unfortunately, as impressive as these molecular glues were in impeding cancer cell proliferation in vitro, neither HQ461 nor NCT02 was metabolically stable in vivo, which restricted the use of these drugs in animals. The pharmacokinetics of dCeMM2/3/4 was not examined in vivo.

PP-C8 is a cereblon (CRBN)-recruiting PROTAC with a dual CDK12/13 inhibitor SR-4835-based warhead [83]. In this study, it displayed strong degradation capabilities against Cyclin K and CDK12, without affecting the protein level of CDK13. Although the effect of PP-C8 on cancer cells was similar to Cyclin K or CDK12 depletion, the in vivo metabolic stability of this drug was not examined. Notably, 7f and 7b (abbreviated as 7f/7b) are also CRBN-recruiting PROTACs sharing the same warhead and E3 ligand, although they differ in the linker region [82]. The warhead, also known as the ligand for the protein of interest, was designed based on a previously reported dual CDK12/13 inhibitor compound “2” [87]. Global proteomic profiling showed that Cyclin K, CDK12, and CDK13 were among the top proteins degraded by 7f [82]. The 7f-mediated degradation of CDK12 and CDK13 was validated in vitro. Additionally, 7b was shown to further degrade CDK12 and CDK13 in vivo.

SR4835 is a dual CDK12/13 inhibitor that exhibited strong degradation activity against Cyclin K [57,80]. In these studies, it was shown to be functionally different from another covalent CDK12/13 inhibitor, THZ531, which only inhibited the kinase activity of CDK12/13 but failed to affect the protein level of Cyclin K [80,88]. SR4835 was used in mouse models in several studies to investigate the role of Cyclin K, CDK12, or CDK13 in cancers [57,68,80,85]. However, the way in which SR4835 triggers the degradation of Cyclin K is unclear. CR8 was first reported in 2008 as a roscovitine-derived pan-CDK inhibitor against CDK1/2/5/7/9 [89]. In 2020, it was given another identity as a molecular glue against Cyclin K [25]. CR8 was rediscovered as a molecular glue degrader during the bioinformatic screening of 4518 drugs in a correlation analysis between drug toxicity and the mRNA levels of E3 ligase components across 578 cell lines. The toxicity of CR8 on cells correlated well with the mRNA level of DDB1 when quantitative expression proteomics showed a consistent decrease in Cyclin K abundance via CR8 treatment. The subsequent biochemistry experiments confirmed that CR8 belonged to the thalidomide analog molecular glue and brought CDK12 into proximity with the E3 ligase for Cyclin K ubiquitination and degradation. Although CR8 was proved to be suitable for in vivo studies [90], the use of this drug in Cyclin K/CDK12/CDK13-associated studies was restricted due to the wide range of its CDK targets.

## 5. Concluding Remarks

Cyclin K, as a regulatory subunit of CDK12 and CDK13, controls diverse biological processes by regulating gene transcription, translation, and the key cell signaling pathways. However, during the past two decades, increased efforts have been made to understand the functions of the catalytic subunits CDK12 and CDK13 in cancer biology. The biomedical significance of Cyclin K in cancers has been overlooked for some time. Thanks to breakthroughs in targeted protein degradation technologies, we are now in an exciting era of targeted cancer therapeutics, wherein previously undruggable proteins can be targeted, such as cyclins and other proteins lacking enzymatic or allosteric sites. In recent years, Cyclin K has been garnering increased research attention and is demonstrated as a critical player for cancer growth and therapeutic resistance. Targeting Cyclin K promises an efficient approach because it leads to the inhibition of both CDK12 and CDK13. Better yet, Cyclin K can be targeted by varying therapeutic modalities, indicating its high druggability. Reviewing the previous studies published on CDK12, the therapeutic potential and treatment avenues involving CDK12 can quickly be realized. After determining Cyclin K to be the binding partner for CDK12/13, it has been only 10 years since researchers have begun to understand CDK12 well enough to utilize it as a biomarker for immunotherapy and PARPi in the clinical trials, which can be considered “better late than never”. 

The present research climate is promising for increasing our knowledge of Cyclin K to fight various cancers. The following examples are some of the interesting aspects for consideration in future Cyclin K-centered cancer studies. 

First, understanding the genomic pattern and epigenomic control of Cyclin K will help us identify the mutation pattern of Cyclin K, the associated genetic alterations, and the underlying mechanisms for Cyclin K overexpression in cancers. These findings may provide the necessary information to answer some important questions, such as whether Cyclin K can be used as a biomarker, or whether there is a therapeutic benefit for epigenetic-targeted therapy in Cyclin K-overexpressed tumors. 

Second, exploring the involvement of Cyclin K in immuno-oncology is expected to elucidate key information. Immunotherapy is an active area in cancer research and is advantageous compared to traditional therapies in certain ways, such as its high effectivity, reduced relapse risk, less toxicity, etc. Current studies implicate the potential participation of Cyclin K in cancer immunotherapy. More work is needed to determine whether Cyclin K is indeed a master regulator for immune suppression in cancers. 

Third, investigating the function of Cyclin K in cancer development using genetically engineered mouse models is another way to increase our understanding of its role in cancer progression. To generate a more profound understanding of how Cyclin K contributes to cancer development, there is a need for improved mouse models. The constitutive Cyclin K-knockout mouse cannot survive beyond embryonic development. Alternatively, tissue-specific knockout mouse models or inducible transgenic mouse models will have a strong potential to offer a greater chance of animal survival and can provide more details on the role of Cyclin K in disease control and tissue development. 

Fourth, an avenue of research that is still underexplored is the discovery or design of improved Cyclin K-targeting drugs. Despite the availability of several Cyclin K-degrading drugs, many drugs are metabolically unstable in vivo or have not yet been examined in vivo. To advance bench-to-bedside transition, Cyclin K-targeting drugs with good pharmacokinetics and pharmacodynamics are needed. Moreover, current Cyclin K degraders are capable of targeting CDKs simultaneously. Developing Cyclin K-specific degraders represents a promising approach that may be less toxic to healthy tissues in vivo.

Considering how much we have achieved in improving the survival rate for cancer patients, some cancers are still very difficult to detect and are refractory to treatments. More research is needed to understand the molecular mechanisms of cancer and develop improved tools for cancer diagnosis and therapies. Given the great therapeutic potential of CDKs and cyclins, as well as rapid advancements in drug discovery and development, it is a prime time to scrutinize the involvement of Cyclin K in cancers and develop effective drugs against Cyclin K. Ideally, Cyclin K-targeting therapies will be used as powerful anti-cancer arsenals in the future and, one day, we can turn the devastation of cancer into a manageable chronic disease.

## Figures and Tables

**Table 1:** CDK inhibitors in clinical trials for interventional cancer studies (as of 18 May 2023).

CDK Targets	Name of the Inhibitors	Indications in Cancers	Clinical Phases
CDK4/6	Palbociclib (PD0332991), ribociclib (LEE011), abemaciclib (LY2835219), trilaciclib (G1T28), dalpiciclib (SHR6390), lerociclib (G1T38, GB491), SPH4336, TQB3616, TQB3303, XZP-3287, HS-10342, PRT3645, FCN-437c, BPI-1178, GLR2007, and CS3002	Breast cancer, non-small cell lung cancer, small-cell lung cancer, prostate cancer, glioma, liposarcoma, ovarian cancer, head and neck squamous cell carcinoma, colorectal cancer, melanoma, pancreatic cancer, endometrial cancer, acute lymphoblastic leukemia, multiple myeloma, and other advanced malignancies	I, II, III
CDK4	PF-07220060	Breast cancer, lung cancer, prostate cancer, liposarcoma, and colorectal cancer	I, II
CDK4/6 and FLT3	FLX925	Acute myeloid leukemia	I
CDK4/6, Pim-3, and FLT3	ETH-155008	Non-Hodgkin’s lymphoma and acute myeloid leukemia	I
CDK2	PF-07104091, INX-315, and INCB123667	Breast cancer, ovarian cancer, and small cell lung cancer	I, II
CDK7	Samuraciclib (CT7001), SY-5609, Q901, and XL102	Breast cancer, small cell lung cancer, prostate cancer, ovarian cancer, colorectal cancer, and pancreatic cancer	I, II
CDK9	Enitociclib (VIP152, BAY1251152), AZD4573, KB-0742, and GFH009	Non-Hodgkin’s lymphoma, Hodgkin’s lymphoma, multiple myeloma, and refractory solid tumors	I, II
CDK1/2/3/4/7/9	Roniciclib (BAY1000394)	Non-small-cell lung cancer, small-cell lung cancer, and other advanced malignancies	I, II
CDK1/2/4	AG-024322	Advanced malignancies	I
CDK1/2/4/9	Riviciclib hydrochloride (P276-00)	Breast cancer, head and neck squamous cell carcinoma, melanoma, pancreatic cancer, multiple myeloma, mantle cell lymphoma, and other advanced malignancies	I, II
CDK1/2/4/5/9	AT7519M	Non-Hodgkin’s lymphoma, multiple myeloma, and advanced solid tumors	I, II
CDK1/2/4/6/9	Alvocidib (flavopiridol, L86-8275), TP-1287 (prodrug of alvocidib)	Non-Hodgkin’s lymphoma, acute myeloid leukemia, multiple myeloma, myelodysplastic syndrome, Ewing sarcoma, synovial sarcoma, liposarcoma, pancreatic cancer, ovarian cancer, endometrial cancer, head and neck squamous cell carcinoma, melanoma, and other advanced malignancies	I, II
CDK1/2/5/9	Dinaciclib (SCH-727965)	Breast cancer, melanoma, pancreatic cancer, non-small-cell lung cancer, non-Hodgkin’s lymphoma, acute myeloid leukemia, multiple myeloma, and other advanced malignancies	I, II, III
CDK1/4/6/9	Voruciclib (P1446A-05)	Melanoma, acute myeloid leukemia, and other advanced malignancies	I
CDK2/4/6	RGT-419B, PF-06873600, NUV-422	Breast cancer, prostate cancer, glioma, fallopian tube cancer, and peritoneal cancer	I, II
CDK2/7/9	SNS-032 (BMS-387032)	Non-Hodgkin’s lymphoma, multiple myeloma, and advanced solid tumors	I
CDK2/9	Fadraciclib (CYC065)	Acute myeloid leukemia, myelodysplastic syndrome, non-Hodgkin’s lymphoma, breast cancer, colorectal cancer, ovarian cancer, endometrial cancer, hepatocellular carcinoma, and biliary tract cancer	I, II
CDK1/2/9, JAK2, and FLT3	Zotiraciclib (SB1317, TG02), zotiraciclib citrate	Astrocytoma, astroglioma, glioblastoma, gliosarcoma, hepatocellular carcinoma, colorectal cancer, non-Hodgkin’s lymphoma, acute myeloid leukemia, multiple myeloma, myelodysplastic syndrome, and blast crisis	I, II
CDK8/19	RVU120 (SEL120), BCD-115	Acute myeloid leukemia, myelodysplastic syndrome, breast cancer, and other solid tumors	I, II
CDK8/19 and multiple tyrosine kinases	TSN084	Advanced malignancies	I

**Table 2:** Current Cyclin K-degrading drugs.

Name	Category	Main Degradation Targets	Reference
HQ461	Molecular glue	Cyclin K, CDK12	[86]
NCT02	Molecular glue	Cyclin K, CDK12	[80]
dCeMM2/3/4	Molecular glue	Cyclin K, CDK12, CDK13	[81]
PP-C8	PROTAC	Cyclin K, CDK12	[83]
7f/7b	PROTAC	Cyclin K, CDK12, CDK13	[82]
SR4835	CDK12/13 inhibitor	Cyclin K, CDK12	[80]
CR8	Pan-CDK inhibitor/molecular glue	Cyclin K	[25]

## Data Availability

Not applicable.
